# Exposure of Human Skin Models to KrCl Excimer Lamps: The Impact of Optical Filtering^†^

**DOI:** 10.1111/php.13383

**Published:** 2021-02-05

**Authors:** Manuela Buonanno, David Welch, David J. Brenner

**Affiliations:** ^1^ Center for Radiological Research Columbia University Medical Center New York NY USA

## Abstract

Far‐UVC radiation is a promising technology that is potentially both effective at killing airborne microbes such as coronaviruses and influenza, and being minimally hazardous to the skin and eyes. Our previous studies on health risks from far‐UVC have employed a krypton‐chloride (KrCl) excimer lamp, emitting principally at 222 nm, supplemented with an optical filter to remove longer wavelength emissions inherent to these lamps. This study explores KrCl lamp health hazards by comparing filtered and unfiltered KrCl lamps using effective spectral irradiance calculations and experimental skin exposures. Analysis of effective irradiances showed a notable increase in allowable exposure when using a filter. Induction of DNA dimers (CPD and 6‐4PP) was measured in human skin models exposed to a range of radiant exposures up to 500 mJ cm^−2^. Compared to sham‐exposed tissues, the unfiltered KrCl lamps induced a statistically significant increase in the yield of both DNA lesions at all the radiant exposures studied. Conversely, filtered KrCl lamps do not induce increased levels of dimers at the current daily TLV exposure limit for 222 nm (23 mJ cm^−2^). This work supports the use of filters for far‐UVC KrCl excimer lamps when used to limit disease transmission in occupied locations.

## Graphical abstract

Far‐UVC radiation is a promising tool to reduce the transmission of airborne‐mediated diseases. This study explores the skin health hazards using KrCl excimer lamps as a far‐UVC source. The KrCl lamp was tested with and without added filtration to reduce emissions outside of the 222 nm peak. The filtered KrCl lamp did not produce DNA photodamage for radiant exposures lower than 500 mJ cm^−2^. Conversely, the unfiltered KrCl lamp induced a statistically significant increase in DNA dimers at all the radiant exposures studied, including the current recommended exposure limit for 222 nm radiation of 23 mJ cm^−2^.

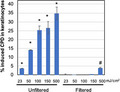

## Introduction

The COVID‐19 pandemic, caused by the SARS‐CoV‐2 virus, highlights the need for new tools to control the spread of diseases. Limiting microbial disease transmission extends to future novel pathogenic diseases as well, especially because vaccines or other treatments are not immediately identified and available.

An increasing body of evidence indicates that far‐UVC radiation in the range of 200‐230 nm is as effective as standard germicidal UV radiation (typically from low‐pressure mercury lamps emitting at 254 nm) at killing a variety of microbial pathogens (1, 2, 3, 4, 5) including drug‐resistant bacteria (6), and at inactivating viruses (1, 7, 8). Specifically, we and others have shown that 222‐nm radiation inactivates influenza A (H1N1) (7) and human coronaviruses (8), including SARS‐CoV‐2 (9).

In addition to its germicidal potential, far‐UVC radiation has been shown to be minimally hazardous for skin and eyes using various experimental models (10, 11, 12, 13) as well as in exposed human volunteers (14). Thus, far‐UVC technology is a promising tool that could be used in occupied indoor public locations such as hospitals and schools to limit the spread of both airborne‐ and surface‐mediated microbial diseases (7, 8, 9).

The far‐UVC source used in this study is an excimer lamp (excilamp) based on a krypton‐chloride (KrCl) gas mixture (15). A KrCl lamp emits primarily at 222 nm, but other emissions outside of this peak extend up through the UVC due to electron transitions other than the transition from the KrCl exciplex (16). Proteins absorb UVC radiation at a rate that is wavelength dependent; specifically, far‐UVC wavelengths are more highly absorbed by proteins than longer wavelength UVC (17). Therefore, longer wavelength emissions, such as those from the nonpeak transitions from KrCl excimer lamps, penetrate further into tissue than far‐UVC emissions. These more penetrating photons can potentially damage the DNA of cells deeper within human tissues. In fact, when phototype I and II skin of healthy volunteers was exposed to a KrCl source that had a ~ 3% component from wavelengths above 250 nm, erythema and DNA photodamage ensued (18, 19). Using Monte Carlo simulations of ultraviolet radiation transport in skin, it was estimated that the contribution to DNA photodamage in the epidermis from wavelengths shorter than 230 nm was negligible (19, 20). With germicidal far‐UVC sources proposed to be used in occupied locations, such findings emphasize the importance of characterization of their emission spectra in order to ensure that the recommended safety exposure limits are not exceeded (21, 22).

In previous 222‐nm safety studies, we (6, 23) and others (12, 13) have consistently used an optical filter to reduce the emissions outside the nominal peak. To highlight the importance of filtering out potential harmful wavelength components emitted by far‐UVC sources, here we compared the induction of cyclobutane pyrimidine dimers (CPD) and 6‐4 pyrimidine pyrimidone dimers (6‐4PP) in a model of human skin exposed to different acute radiant exposures from a filtered or unfiltered 222‐nm KrCl excilamp. Studies comparing the effects of an acute 222 nm or 254 nm exposure at their corresponding daily exposure limit were also performed. These daily exposure limits are published by the American Conference of Governmental Industrial Hygienists (ACGIH) and the International Commission on Non‐Ionizing Radiation Protection (ICNIRP) and are in agreement and also widely accepted (21, 22). We have also included a study on the theoretical exposure limits for both unfiltered and filtered KrCl lamps, again based on the ACGIH and ICNIRP recommendations. This analysis utilizes measured spectral irradiance data and then considers the limits of operating these sources within current guidelines.

## Materials and Methods

### Ultraviolet radiation sources

The primary source used in this work was a krypton‐chloride (KrCl) excimer lamp (High Current Electronics Institute, Tomsk, Russia) which emits principally at 222 nm. The KrCl lamp was also used with the addition of a custom optical filter (Ushio America, Cypress, CA) to reduce the emissions outside the 222 nm peak. Also used in testing was a low‐pressure mercury lamp (Mineralight XX‐15S, UVP, Upland, CA) which principally emits at 254 nm.

### Lamp characterization

**Figure 1:**
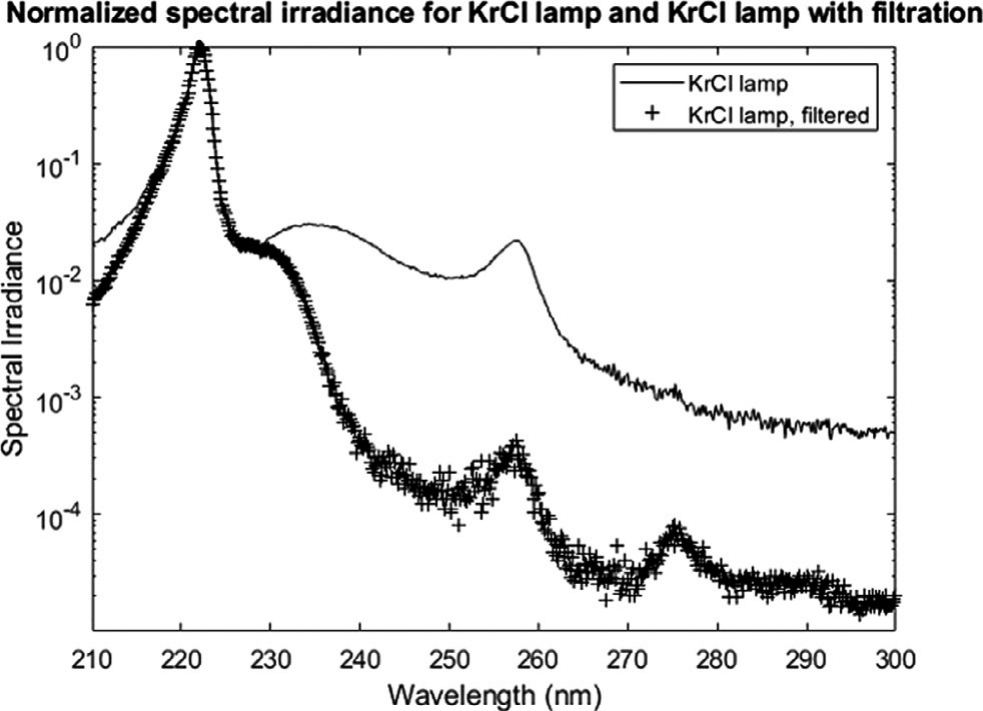
Plot of the spectral irradiance of a KrCl lamp normalized to the 222 nm peak (–). Also plotted is the normalized spectral irradiance of the KrCl lamp with the addition of a custom filter that reduces the intensity outside of the 222 nm peak (+).

An AvaSpec‐ULS4096CL‐EVO spectrometer (Avantes Inc., Louisville, CO) calibrated to measure absolute irradiance was used to obtain the irradiance spectra. The normalized spectral irradiance for the KrCl lamp alone and with the addition of a filter is shown in Fig. 1. The spectral irradiance of the filtered KrCl lamp was plotted by multiplying the unfiltered KrCl spectrum by the transmission of the custom filter. This approach for obtaining the filtered KrCl spectrum was required because the addition of a filter to the KrCl lamp decreased the emissions outside of the 222 nm peak below detectable levels for the spectrometer. In addition to the radiometrically calibrated spectrometer, a calibrated Hamamatsu UV Power Meter (C9536/H9535‐222, Hamamatsu Photonics K.K., Japan) was also used to verify the irradiance values during exposures. Exposures for the unfiltered KrCl lamp were performed with an irradiance of 0.85 mW cm^−2^, and exposures with the filtered KrCl lamp used an irradiance of 0.59 mW cm^−2^. The irradiance of the low‐pressure mercury lamp was 0.24 mW cm^−2^.

### Effective irradiance calculations

The ICNIRP and ACGIH have published guidelines on the monochromatic exposure limits for all wavelengths of ultraviolet radiation over an 8‐h period. Included in these guidelines are the spectral weighting factors for specific wavelengths (21, 22), which reflects the relative hazard of each wavelength relative to the peak hazard at 270 nm. We have applied the ICNIRP spectral weighting factors to the normalized spectral irradiance data utilized in this study, shown in Fig. 1, to determine the effective irradiance (*E*_eff_) of each spectrum used in this work. The total area under each normalized spectral irradiance curve was calculated, as was the area under the effective spectral irradiance curves. The total effective spectral hazard of a lamp, *S*(lamp), is simply the ratio of the total effective irradiance (*E*_eff_) to the normalized irradiance:

(1) $$S(\text{lamp})=\frac{E_{\text{eff}}\;\mathrm{Normalised}\;\text{Area}}{\mathrm{Normalised}\;\text{Area}}$$

The exposure limit for a broadband source is then determined *b* dividing the 3 mJ cm^−2^ daily radiant exposure limit by *S*(lamp) for that source:

$\text{Lamp Exposure Limit}=\frac{3\frac{\text{mJ}}{{\text{cm}}^{2}}}{S(\text{lamp})}$ (2)

We note that normalization of the spectral irradiance is not required for these calculations but is performed in this work to allow for an easier visual comparison of the relative contributions of the nonpeak emissions seen in the filtered and unfiltered KrCl sources. Normalization has no effect on the calculated hazard value, *S*(lamp), or the lamp exposure limit.

### UV‐induced premutagenic DNA lesions in a 3‐D human skin model

We used the 3‐dimensional (3‐D) human skin model EpiDerm‐FT (MatTek Corp., Ashland, MA) consisting of stratum corneum and 8‐12 human cell layers to reproduce human epidermis and dermis (24). We measured induction of the two most abundant premutagenic DNA lesions, cyclobutane pyrimidine dimers (CPD) and pyrimidine‐pyrimidone 6‐4 photoproducts (6‐4PP), in keratinocytes of the epidermis (25) as a function of the UVC radiant exposure. Sham and exposed tissues were fixed 15 min after exposure, and the DNA photoproducts were detected using the immunohistochemical method previously described (26).

### Statistical analysis

DNA photodamage yields represent the average ± standard error of the mean (SEM) of keratinocytes exhibiting dimers measured in at least six randomly selected fields of view for each sample (*n* = 2 or 3). For each sample, an average of approximately 1470 cells was counted for CPD assessment and 1320 cells were counted for 6‐4PP assessment. Comparisons of mean values between treatment groups and controls were performed using Student's t‐test.

## Results

### Effective irradiance calculations

**Figure 2:**
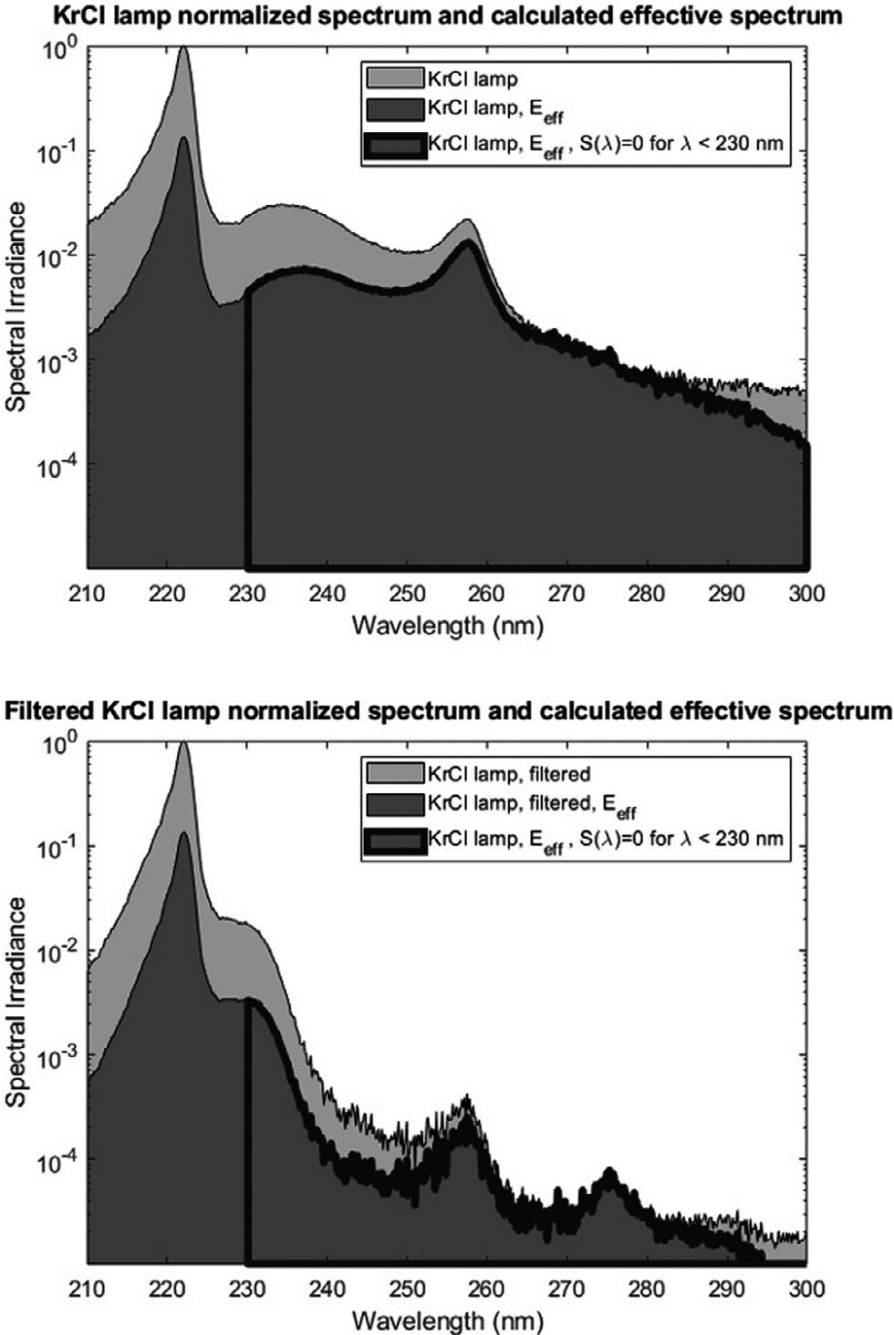
Normalized spectral irradiance of an unfiltered (top) and filtered (bottom) KrCl lamp along with the calculated effective spectrum. Also included on each plot are the effective spectral areas used for the hypothetical situation where the hazard function *S*(*λ*) has a value of zero for wavelengths below 230 nm.

We have applied the ICNIRP equations (22) to the normalized spectral irradiance data shown in Fig. 1 to determine the effective irradiance of each spectrum used in this work. Each of these effective irradiance plots is shown in Fig. 2. Results from the calculations of effective irradiance for the filtered and unfiltered KrCl lamp are provided in Table 1. Additionally, the hazard for the low‐pressure mercury lamp was calculated to be *S* = 0.49.

**Table 1:** Calculated lamp exposure limits using measured and calculated spectral irradiance data shown in Fig. 2. The area under the spectral irradiance plots is used to find the total spectral effectiveness, *S*(lamp), for a given spectrum. The calculated exposure limit for the KrCl lamp both with and without added filtration is also given. Calculated values for the hypothetical situation of zero hazard (or relative spectral effectiveness *S*(*λ*) = 0) for wavelengths lower than 230 nm are also provided.

	Area under curve	$S\left(\text{lamp}\right)=\frac{E_{\text{eff}}\;\text{Area}}{\text{Area}}$	$\text{Lamp Exposure Limit}=\frac{3\;{\text{mJ/cm}}^{2}}{S(\text{lamp})}$
KrCl lamp	3.75		
KrCl lamp, *E*_eff_	0.642	0.171	17.5 mJ cm^−2^
KrCl lamp, *E*_eff_ with *S*(*λ*) = 0 for *λ* < 230 nm	0.237	0.063	47.6 mJ cm^−2^
Filtered KrCl lamp	3.03		
Filtered KrCl lamp, *E*_eff_	0.404	0.133	22.5 mJ cm^−2^
Filtered KrCl lamp, *E*_eff_ with *S*(*λ*) = 0 for *λ* < 230 nm	0.0157	0.005	600 mJ cm^−2^

While the KrCl lamp emits primarily at 222 nm, the calculated exposure limit for the unfiltered lamp is only 17.5 mJ cm^−2^, which is about 76% of the allowable 23 mJ cm^−2^ exposure limit if the lamp only emitted at 222 nm. The addition of the filter raises the exposure daily limit to 22.5 mJ cm^−2^, or almost 98% of the allowable exposure if the lamp only emitted at 222 nm.

The necessity of filtering appears even more crucial when considering the future outlook of far‐UVC implementations. Considering the body of work from our laboratory and others showing the relative low health risk of far‐UVC exposures, it is possible that exposure limit recommendations will be reconsidered by regulating bodies and possibly amended. However, the magnitude and extent of these changes are currently unknown. As an exercise, we have examined hypothetical effects from raising limits for far‐UVC exposure and how this would impact the calculation of effective irradiance from KrCl sources with and without filtering. We have chosen to look at an extreme case where the hazard for all wavelengths less than 230 nm is reduced to zero. Zero hazard is not realistic, as all wavelengths throughout the UV have an associated hazard value published (22), but for illustrative purposes, this approach provides interesting results. The calculated effective irradiances for both the unfiltered and filtered KrCl lamp with this adjustment to *S*(*λ*) are included in Table 1 and Fig. 2. Importantly, even with a hypothetical zero hazard from wavelengths <230 nm, the maximum allowable dose from the unfiltered KrCl lamp is only about 47.6 mJ cm^−2^. Therefore, even if the exposure limit for 222 nm radiation was adjusted to a value above a radiant exposure of 47.6 mJ cm^−2^, this dose could never be applied with an unfiltered lamp while staying within the total exposure limit. Performing the same calculation on the filtered KrCl lamp yields a maximum possible radiant exposure of 600 mJ cm^−2^. This 13‐fold increase in permissible exposure limit is due to the decrease in nonpeak emissions through optical filtering and is clearly important for utilizing any significant changes to recommended exposure limits in the far‐UVC.

### Induction of premutagenic DNA lesions in human skin models

**Figure 3:**
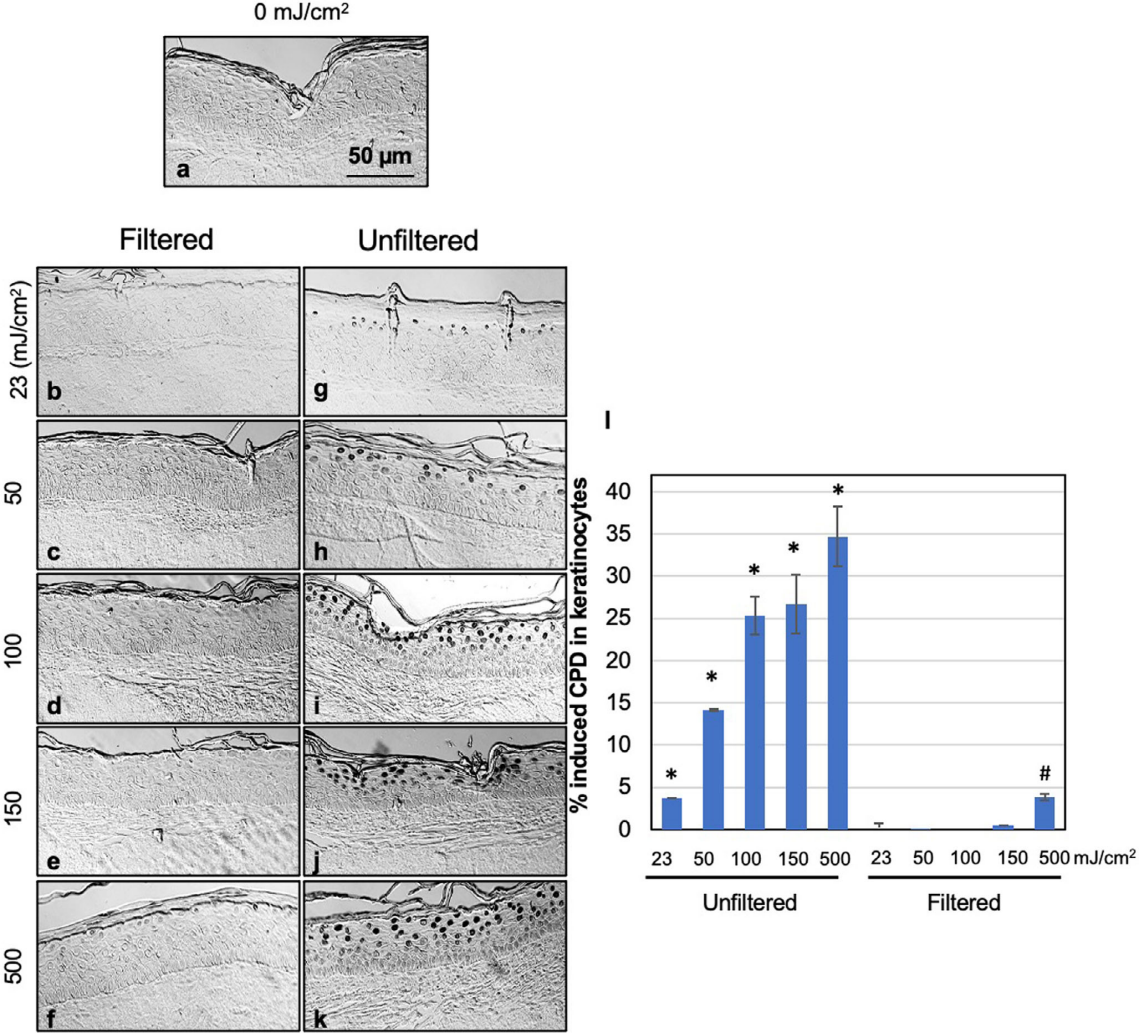
Cyclobutane pyrimidine dimers (CPD) yields induced by filtered and unfiltered 222‐nm UV radiation in 3‐D human skin tissue models. Representative cross‐sectional images of tissue models comparing premutagenic skin lesions CPD (dark‐stained cells) in the epidermis of sham‐exposed samples (a), of samples exposed to 23, 50, 150, or 500 mJ cm^−2^ from filtered (b‐f) or unfiltered 222‐nm radiation (g–k). (l) Quantification of the percentage of keratinocytes showing CPD dimers. Values represent the average ± SEM of cells exhibiting dimers measured in at least six randomly selected fields of view per samples (*n* = 2 or 3; an average of ~ 1470 cells per sample were counted); *P < 0.001, #*P* < 0.05.

**Figure 4:**
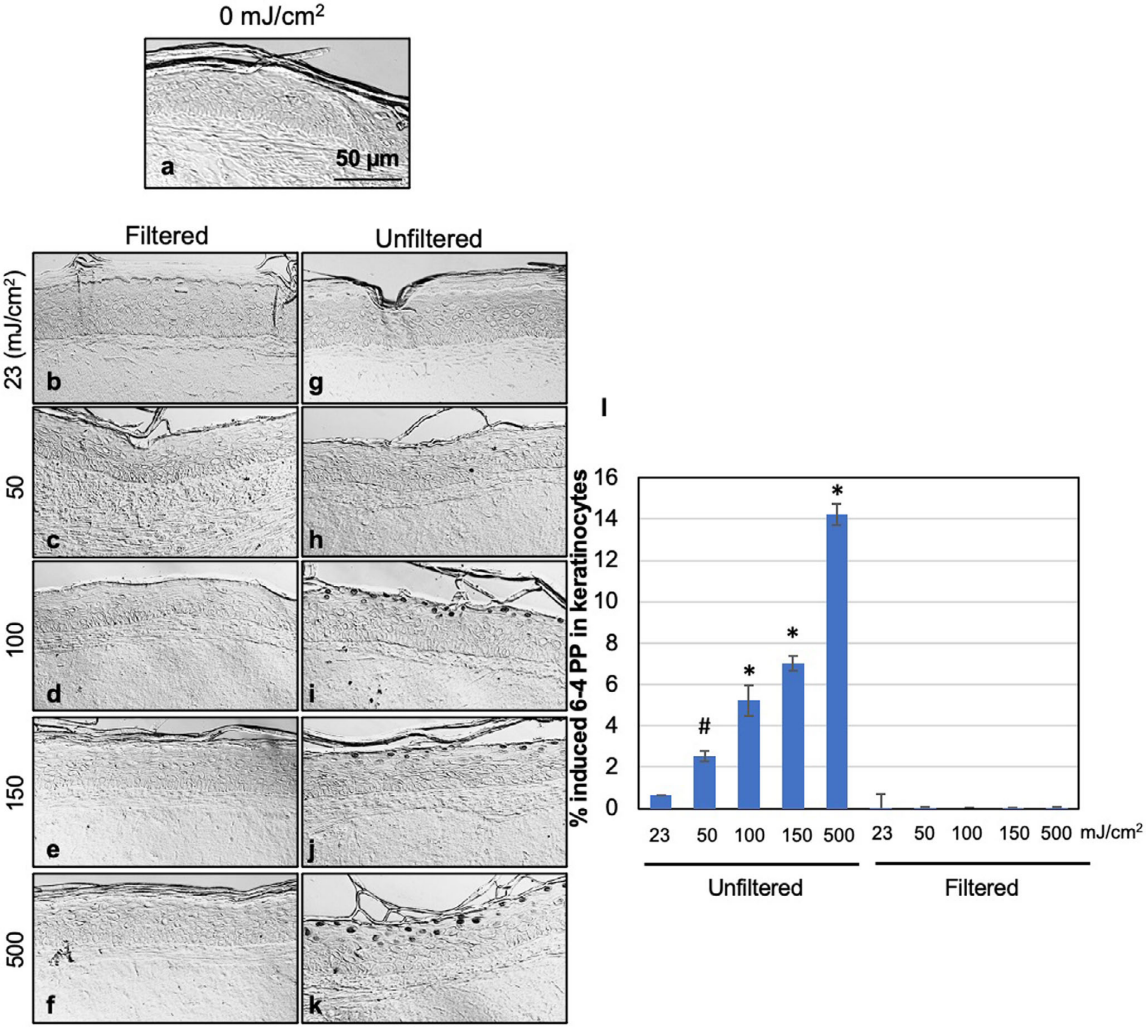
6‐4 pyrimidine pyrimidone dimers (6‐4PP) yields induced by filtered and unfiltered 222‐nm UV radiation in 3‐D human skin tissue models. Representative cross‐sectional images of tissue models comparing premutagenic skin lesions 6‐4PP (dark‐stained cells) in the epidermis of sham‐exposed samples (a), of samples exposed to 23, 50, 150, or 500 mJ cm^−2^ from filtered (b–f) or unfiltered (g–k) 222‐nm radiation. (l) Quantification of the percentage of keratinocytes showing 6‐4PP dimers. Values represent the average ± SEM of cells exhibiting dimers measured in at least six randomly selected fields of view per samples (*n* = 2 or 3; an average of ~ 1320 cells per sample were counted); *P ≤ 0.001, #*P* < 0.05.

Figures 3 and 4 show representative cross‐sectional images of human skin tissue models exposed to 0, 23, 50, 150, or 500 mJ cm^−2^ from filtered (b–f) or unfiltered 222‐nm radiation (g–k), showing the presence of keratinocytes with CPD (Fig. 3) and 6‐4PP dimers (Fig. 4). Instances of dimer formation appear as dark‐stained cells. Compared to controls (Fig. 3a), exposure of tissues to radiant exposures as low as 23 mJ cm^−2^ from the unfiltered 222‐nm radiation (Fig. 3g–l), induced an increase of CPD dimers (*P* < 0.001). In contrast, tested exposures up to 150 mJ cm^−2^ from the filtered 222‐nm radiation did not induce a yield of CPD that was significantly higher than that in the sham‐irradiated samples (Fig. 3a–e,l), in agreement with our previous studies (6, 23). However, tissues exposed to the highest tested radiant exposure from the filtered 222‐nm radiation (500 mJ cm^−2^) (Fig. 3f,l) showed a small but statistically significant increase of CPD relative to unirradiated controls (*P* < 0.05); notably, the CPD‐positive keratinocytes were located mainly in the uppermost epidermal cell layer (Fig. 3f) and never involved the replicating basal cells in the deepest part of epidermis, which play a critical role in the development of skin cancer (27, 28). As discussed above, the KrCl excilamp is not a single wavelength source and the use of the optical filtering reduces but does not completely remove the emission of wavelengths outside of the 222‐nm peak. In addition, the sample exposed to 500 mJ cm^−2^ from the filtered 222‐nm radiation (Fig. 3f) showed a percentage of the induced CPD (3.9 ± 0.5) that was comparable to that measured in tissues exposed to 23 mJ cm^−2^ from the unfiltered lamp (3.8 ± 0.1) (Fig. 3 g).

In the case of 6‐4PP, exposure to the filtered KrCl emissions at any of the studied radiant exposures did not induce yields that were significantly higher than those measured in sham‐irradiated samples (Fig. 4a–f,l). In contrast, tissues exposed to as low as 50 mJ cm^−2^ from the unfiltered KrCl lamp (Fig. 4h–k,l) exhibited a statistically significant increase of induced 6‐4 photoproducts compared to sham‐exposed tissues (*P* < 0.05 or *P* < 0.001).

### DNA photodamage induced by acute exposure limits

The currently recommended exposure limits for an individual during an 8‐h period or TLV (Threshold Limit Values) are 6 mJ cm^−2^ for 254‐nm radiation and 23 mJ cm^−2^ for 222‐nm radiation (21, 22). Using the human skin model, we assessed the induction of DNA photodamage from such radiant exposures delivered acutely.

**Figure 5:**
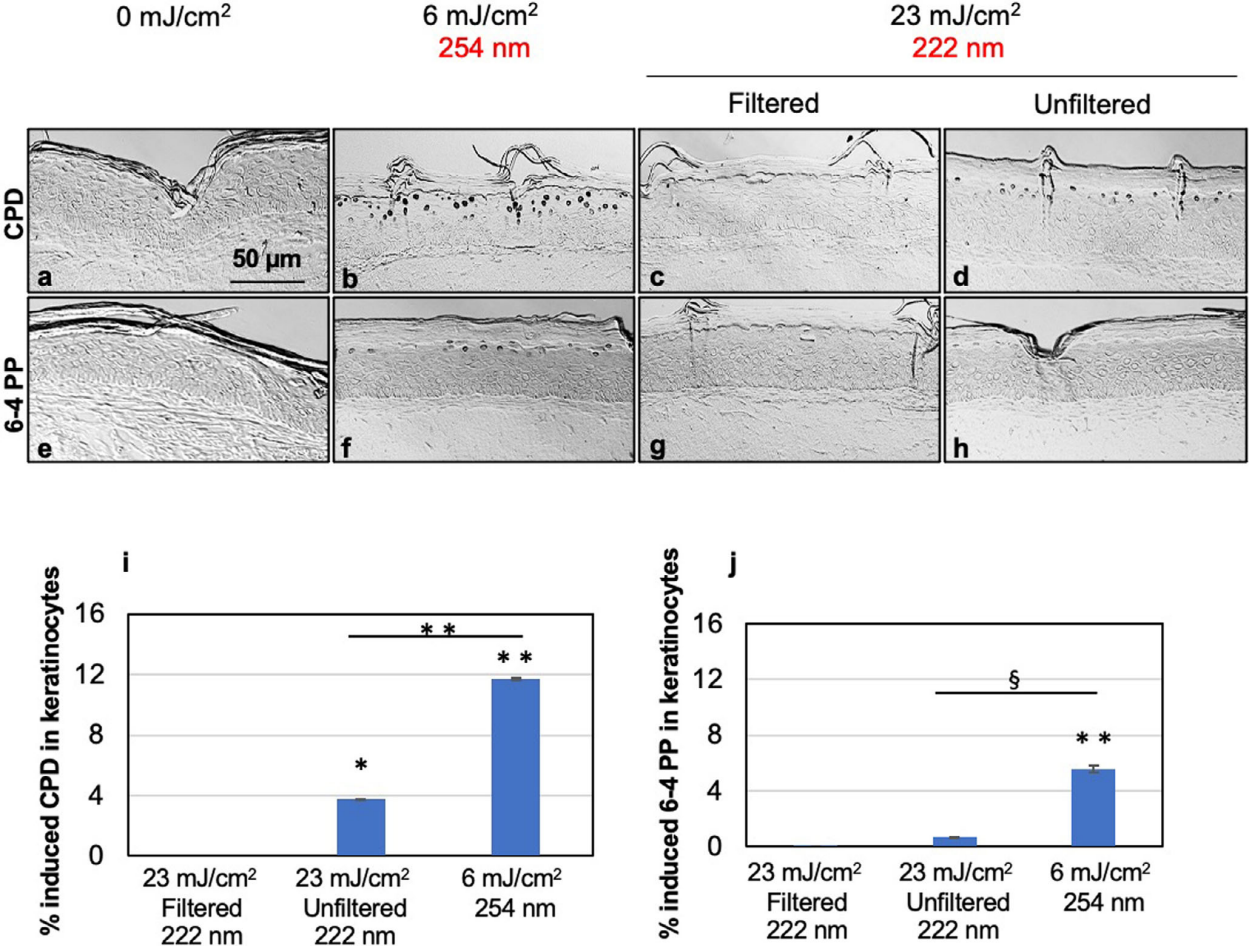
Premutagenic DNA lesion yields induced on 3‐D human skin tissues by 222‐ or 254‐nm UV radiation at the corresponding current regulatory dose limits. Representative cross‐sectional images of samples comparing premutagenic skin lesions CPD ((a–d), dark‐stained cells), and 6‐4PP ((e‐h), dark‐stained cells) in the epidermis of sham‐exposed skin ((a) for CPD and (e) for 6‐4PP); of samples exposed 6 mJ cm^−2^ from 254‐nm radiation ((b) for CPD and (f) for 6‐4PP); of samples exposed to 23 or mJ cm^−2^ from filtered ((c) for CPD and (g) for 6‐4PP) or unfiltered 222‐nm radiation ((d) for CPD and (h) for 6‐4PP). Quantification of the percentage of keratinocytes showing CPD (i) and 6‐4PP dimers (j). Values represent the average ± SEM of cells exhibiting dimers measured in at least six randomly selected fields of view per mouse (*n* = 2; an average of ~ 1335 and 1170 cells per sample were counted for CPD and 6‐4PP, respectively); ^§^ *P* < 0.005, *P < 0.001, **P ≤ 0.0001.

Figure 5 shows typical cross‐sectional images of skin comparing keratinocytes with CPD (a–d) and 6‐4PP (e–h), again appearing as dark‐stained cells, in the epidermis of sham‐exposed skin ((a) for CPD and (e) for 6‐4PP), of samples exposed to 6 mJ cm^−2^ from 254‐nm radiation ((b) for CPD and (f) for 6‐4PP) or to 23 mJ cm^−2^ from filtered ((c) for CPD and (g) for 6‐4PP) or unfiltered 222‐nm radiation ((d) for CPD and (h) for 6‐4PP). Quantification of the percentage of keratinocytes showing CPD (i) and 6‐4PP (j) revealed that 6 mJ cm^−2^ from the 254‐nm radiation induced an elevated number of both lesions compared to sham‐exposed tissues (*P* < 0.001) as well as to tissue exposed to 23 mJ cm^−2^ from both filtered and unfiltered 222‐nm radiation (*P* = 0.0001 for CPD and *P* < 0.005 for 6‐4PP). In addition, compared to the DNA lesions detected in keratinocytes of tissues exposed to 23 mJ cm^−2^ from the unfiltered 222‐nm lamp, skin subjected to an acute exposure of 6 mJ cm^−2^ from 254‐nm radiation showed three times and almost nine times higher percentage of CPD (11.69 ± 0.02 vs. 3.76 ± 0.09) and 6‐4PP (5.59 ± 0.05 vs. 0.64 ± 0.26), respectively. When the 222‐nm source was filtered, the percentages were ~ 730 times and ~ 112 times higher for CPD (11.69 ± 0.02 vs 0.016 ± 0.001) and 6‐4PP (5.59 ± 0.05 vs 0.05 ± 0.04), respectively.

## Discussion

The 222‐nm radiation generated by filtered KrCl excimer lamps has been shown to be minimally hazardous for skin and eye exposure (10, 11, 12, 13, 14) and on that basis could potentially be used continuously in occupied indoor spaces to reduce the transmission of surface and, particularly, airborne‐mediated diseases such as COVID‐19 and influenza. However, the emission spectrum of unfiltered KrCl lamps has a main peak at 222 nm and a small component from longer wavelengths. Due to their increased penetration (17), such off‐nominal wavelengths could potentially traverse and damage the DNA of human cells. As we demonstrated here, it is incorrect to presume the TLV of a lamp by considering only the peak of the spectral output. Such assumptions can result in incorrect calculations of the effective hazard of a lamp since stray emissions can greatly impact the total hazard. Prior to installation of any radiation source where human exposure can occur, a full spectral analysis and hazard calculation should be performed.

Using a 3‐D human skin model and DNA photodamage as the end point, we have shown here that when the longer wavelengths are filtered out, no significant levels of CPD or 6‐4PP DNA damage are induced in skin exposed in the tested conditions of 23 mJ cm^−2^ (the current daily TLV), nor at 50, 100, or 150 mJ cm^−2^ (Figs. 3 and 4). The small but statistically significant induction of CPD (but not 6‐4PP) in the uppermost layer of the epidermis of skin tissues exposed to 500 mJ cm^−2^ from the filtered lamp (Fig. 3f) would suggest a skin TLV at 222 nm that is in between 150 and 500 mJ cm^−2^, a suggestion that is consistent with the recent Notice of Intended Change (NIC) the UVC TLV that was recently published by the ACGIH (29).

Overall, this work supports the use of filtered KrCl excimer lamps for the application of far‐UVC radiation in occupied locations where direct exposure of humans is possible.

## Conflict of Interest

The authors declare the following pending patent: Patent Title: “Apparatus, method and system for selectively affecting and/or killing a virus”. Applicant: The Trustees of Columbia University in the City of New York. Inventors: Gerhard Randers‐Pehrson, David Jonathan Brenner, Alan Bigelow. Application #: US20180169279A1. Aspect of manuscript covered in patent application: Spectrum filtering elements such as multilayer dielectric filters or chemical filters are used to remove unwanted wavelengths, or those wavelengths that can be outside of the preferable range of wavelengths. URL: https://patents.google.com/patent/US20180169279A1/en?oq=20200085984
